# ATF5 and HIF1α cooperatively activate HIF1 signaling pathway in esophageal cancer

**DOI:** 10.1186/s12964-021-00734-x

**Published:** 2021-05-12

**Authors:** Feng He, Hang Xiao, Yixin Cai, Ni Zhang

**Affiliations:** Department of Thoracic Surgery, Tongji Hospital, Tongji Medical College Huazhong University of Science and Technology, Wuhan, 430030 China

**Keywords:** ATF5, HIF1, Esophageal cancer

## Abstract

**Background:**

Esophageal cancer (ESCA) is one of the most common cancers worldwide and has a very poor prognosis. Hypoxia-inducible factor 1 (HIF1) signaling pathway plays a critical role in tumorigenesis and is therefore considered a potential therapeutic target in the treatment of many cancers. Activating transcription factor 5 (ATF5) facilitates the expression of various genes and has been extensively studied for its potential role in cancer treatment.

**Methods:**

The expression level of ATF5 in clinic sample was detected by quantitative real time PCR and immunohistochemistry. ATF5 biological function was investigated by western blot, cell cycle analysis, cell viability assay, luciferase reporter assays, colony formation assay, transwell assay, wound healing assay, tube formation assay, and ELISA assay. CHIP and Re-CHIP assay, GST-pulldown, and RNA-sequencing were used to study the cross-talks between ATF5 and HIF1 complex. Mouse xenograft study was utilized to study the correlation of ATF5 and tumor growth in vivo. Student’s t-test or Chi-square test was used for statistical analysis.

**Results:**

Here, we first found ATF5 was dramatically upregulated in ESCA cancer and related with poor survival time. Next, we found that the expression level of ATF5 had a positive relationship with the proliferation, migration, and invasion ability of ESCA cells. Besides, we innovatively found that ATF5 functions as a novel coactivator in HIF1 transcription complex by binding to HIF1α. Further, we demonstrated that silencing ATF5 phenocopies HIF1α knockdown in tumorigenic properties in vitro and inhibited ESCA tumor angiogenesis and proliferation in vivo.

**Conclusion:**

Herein, we found ATF5 as a novel component of the HIF1 transcription complex. The findings of the present study may provide new insights into the development of a novel and more efficient therapeutic strategy against ESCA.

**Video abstract**

**Supplementary Information:**

The online version contains supplementary material available at 10.1186/s12964-021-00734-x.

## Introduction

Globally, esophageal cancer (ESCA) is the eighth most common cancer, which contributes to the sixth leading cancer-related deaths. Because of its aggressive nature, ESCA is one of the most challenging cancers to treat. Besides, the 5-year survival rate of ESCA is just about 15–25% [1–3]. This low survival rate has not improved despite progress in diagnosis and treatment of the condition in the last decade. Given this, there is still a need to conduct more studies to help in the developments of more effective therapeutic strategies.

Hypoxia-inducible factor 1 (HIF1) is composed of HIF-1α and HIF-1β [4, 5]. Instead of conservatively expressing HIF1β,various signaling pathways can regulate the expression of HIF1α, including PI3K pathways and MAPK pathway [6]. Hypoxia is common in many different solid tumors, including ESCA. In normoxia, HIF1α can be prolyl-hydroxylated, and prolyl-hydroxylation of HIF1α is further regulated by various E3 ubiquitin protein ligases [7]. Previous studies have revealed that HIF1 recruits CBP, p300, and POL II in the process of activating gene transcription [6]. Also, STAT3 has been reported to be another subunit of HIF1 transcriptional complex [8]. The proteins transcriptionally activated by HIF1 transcriptional complex are related with tumor angiogenesis, proliferation, and metastasis [9, 10]. Numerous studies have explored the potential of targeting the HIF1 signaling pathway in cancer treatment, particularly targeting a core HIF1 downstream molecule, vascular endothelial growth factor (VEGF) [4, 11–13]. However, the main challenge has been drug-resistance [14, 15].

ATF5 is a member of the cAMP response element binding protein/activating transcription factor family [16, 17]. ATF5 consists of an amphipathic leucine zipper, which mediates hetero- and homo-dimerization, and an N-terminal portion that combines with DNA [18]. ATF5 can interact with various proteins, such as C/EBPβ, P300, and HSP70, which in-turn transcriptionally activates downstream genes or stabilizes itself [19–21]. ATF5 plays a curial role in the process of cell proliferation, survival, and differentiation [20, 22]. Besides, ATF5 plays a vital role in tumorigenesis of several cancers, including breast cancer, lung cancer, ovarian cancer, pancreatic cancer, rectal cancer, renal cell cancer, hepatocellular cancer [23]. Many studies have examined the effectiveness of anti-ATF5 in cancer treatment [18, 23, 24]. However, the biological functions of ATF5 in ESCA remains unclear, thus evaluating the effect of anti-ATF5 in ESCA treatment has been challenging.

Herein, we first revealed that ATF5 is increased in ESCA and that its overexpression has a negative relationship with the survival time of patients. Besides, ATF5 can increase proliferation, migration, and invasion ability of ESCA cell lines. Further, ATF5 acts as a novel component of HIF1 transcriptional complex by interacting with HIF1α, which together activate the HIF1 signaling pathway. Finally, we demonstrated that anti-ATF5 treatment is effective in inhibiting the progression of ESCA. The findings of the present study indicate that ATF5 can act as a novel therapeutic target in ESCA treatment.

## Materials and methods

### Cell lines, antibodies, and reagents

The cell lines HEK293T were got from the American Type Culture Collection (ATCC, Manassas, VA, USA). The cell lines KYSE30 and OE19 were obtained from fenghbio (Hunan, China). Antibodies for ATF5 (ab184923), VEGFA (ab52917), TIMP1 (ab61224), TWIST1 (ab175430), EGF (ab206423), CD31 (ab134168) and POL II (ab264350) were bought from Abcam (Cambridge, MA, USA). Antibodies for PDK1 (13037), PGK1 (68540), CA9 (5649), CBP (7389), P300 (54062), HIF1α (14179), HIF1β (5537) and flag-tag (2368) were procured from Cell Signaling Technology. The human VEGFA ELISA Kit (EK0539) was purchased from BOSTER Biological Technology (Wuhan, China). Identified primers were synthesized by TSINGKE Biological Technology (Beijing, China). For hypoxic conditions, cells were cultured in a sealed hypoxia chamber with a mixture of 1% O2, 5% CO2, and 94% N2 for 2 h.

### Esophageal cancer samples

All clinic samples were obtained from ESCA patients who did not receive chemotherapy or radiotherapy before surgery at Wuhan Tongji Hospital. The written informed consent of all patients was received. This study was approved by the Huazhong University of Science and Technology Ethics Committee.

### Quantitative real time PCR

Total RNA was isolated using the TRIzol (Invitrogen, Carlsbad, CA, USA). The cDNA was synthesized by SuperScript II reverse transcriptase (Thermo Fisher Scientific). Real-time PCR was conducted using the Real-Time PCR Detection System (Bio-Rad, Hercules, USA). The identified primer sequences are showed in Additional file 1: Table S1.

### Construction of lentivirus

The three ATF5 shRNAs and the control RNA packed lentivirus were purchased from GEN-ECHEM (Shanghai, China). The sequences of shRNA were as followed: 5′-GCGCGAGATCCAGTACGTC-3′; 5′-TGGCTCGTAGACTATGGGA-3′; 5′-ATGTCTATGCCCGTCACAT-3′.

### Immunohistochemistry (IHC)

Immunohistochemistry was conducted using the methods described previously [14]. Two pathologists independently evaluated immunostaining results.

### Cell viability assay

Cell viability was investigated by CCK8 analysis. Cells were seeded into 96-well plates (2 × 10^3^ cells per well). Next, 10 μl CCK8 was added to the cells when necessary, then incubated at 37 °C for 2 h. Finally, cell viability was evaluated using a microplate reader at 450 nm.

### Cell cycle analysis

Selected cells were first washed with cold phosphate-buffered saline (PBS) three times then fixed in 80% ethanol at − 20 °C overnight. Then, cells were cultured with propidium iodide (PI) at room temperature for 5–10 min. Cell cycle distribution was investigated using the Becton–Dickinson FACScan System (Franklin Lakes, NJ, USA).

### Luciferase reporter assays

Pretreated ESCA cells were seeded onto 24-well plates and transfected with following steps for 24 h. Relative luciferase activities were measured using the Dual-Luciferase Assay System (Promega).

### Colony formation assay

Briefly, 500 cells are added into 6-well plates. The culture medium is replaced every three days. After approximately two weeks, image for colonies is gained using a microscope.

### Transwell assay

For the transwell assays, 2 × 10^4^ (migration) or 5 × 10^5^ (invasion) cells with serum-free media were seeded into the upper chamber. The lower chamber was filled with 500 μl medium plus 10% FBS. After 10–16 h, cells were fixed with 4% formaldehyde for 30 min, and then remove cells remaining on the upper membrane. 0.1% crystal violet was used to stain cells remaining on the lower membrane. Image for colonies is gained using a microscope.

### Wound healing assay

A linear wound was made using a 200-μm sterile plastic pipette tip when cells were grown to 95% confluency, 24 h after culturing with mitomycin C (10 μg/ml). Cells were washed three times using PBS. Then, distance of wounds was measured.

### Western blot analysis

Protein was extracted by lysing cells with NP-40 buffer plus 1% protease and phosphatase inhibitors. The concentration of the protein was investigated using BCA assay kit (Thermo Fisher Scientific). Proteins from cell lysates were denatured at 100 °C for 5–10 min. Next, the proteins were separated by SDS-PAGE and then transferred to PVDF membranes. The membranes were incubated with identified primary antibodies at 4 °C overnight, and then were incubated with HRP conjugated secondary antibody. Immunoreactive bands were imaged using ECL regents (Thermo Scientific).

### Tube formation assay

80 μl Matrigel was added into each 96-well plates at 37 ℃ for 30 min. HUVECs (2 × 104 cells), cultured with conditioned media (CM), were added into each well for 12 h. Branching points per field were counted to evaluate the tube formation.

### Enzyme-linked immunosorbent assay (ELISA)

The level of VEGFA in each CM was investigated using an ELISA kit (ab119566) according to the manufacturer’s protocol.

### Chromatin immunoprecipitation (ChIP) and ReCHIP assay

ChIP assay was carried out as described previously [25]. ChIP/ReChIP: ChIP was conducted at first. Binding proteins from CHIP assays were isolated using ReChIP buffer. The complexes were diluted using radio-immunoprecipitation buffer and followed by ChIP using another antibody. The ChIP primers sequences are listed in Additional file 1: Table S2.

### GST pull-down assay

The GST proteins were synthesized using *Escherichia coli*, and then *E. coli was* disrupted using ultrasonic cell and GST-fused proteins were extracted using GTS-beads. The beads were incubated with pure identified proteins and then further washed five times. The protein-binding were further investigated by western blot.

### RNA sequencing (RNA-Seq) and data processing

The mRNA expression level of identified cells was investigated by RNA-seq. Three biological replicates were carried out at the Genergy Biology Company (Shanghai, China) by using HiSeq 3000 (Illumina, USA). Part differentially expressed genes were showed in Additional file 1: Table S4.

### Mouse xenograft studies

Four-week-old male BALB/c nude mice were gained from Beijing Huafukang Bioscience Company. 1 × 10^6^ cells were injected subcutaneously into the left flank. The tumor volume was measured using 0.5 × Length (L) × Width (W) [2]. The mouse xenograft experiment was approved by the Animal Care and Use Committee of Tongji Hospital.

### Statistical analysis

TCGA data was downloaded in http://xena.ucsc.edu/welcome-to-ucsc-xena/; Choosing the top quarter and bottom quarter classified by the expression of ATF5 acted as the high group and low group. Using SangerBox tool to draw Kaplan–Meier plot. Data analysis was carried out using SPSS (version 20.0, SPSS). Statistical significance was investigated using Student’s *t*-test or Chi-square test. *P* < 0.05 was considered statistically significant.

## Results

### ATF5 is upregulated in ESCA

**Fig. 1:**
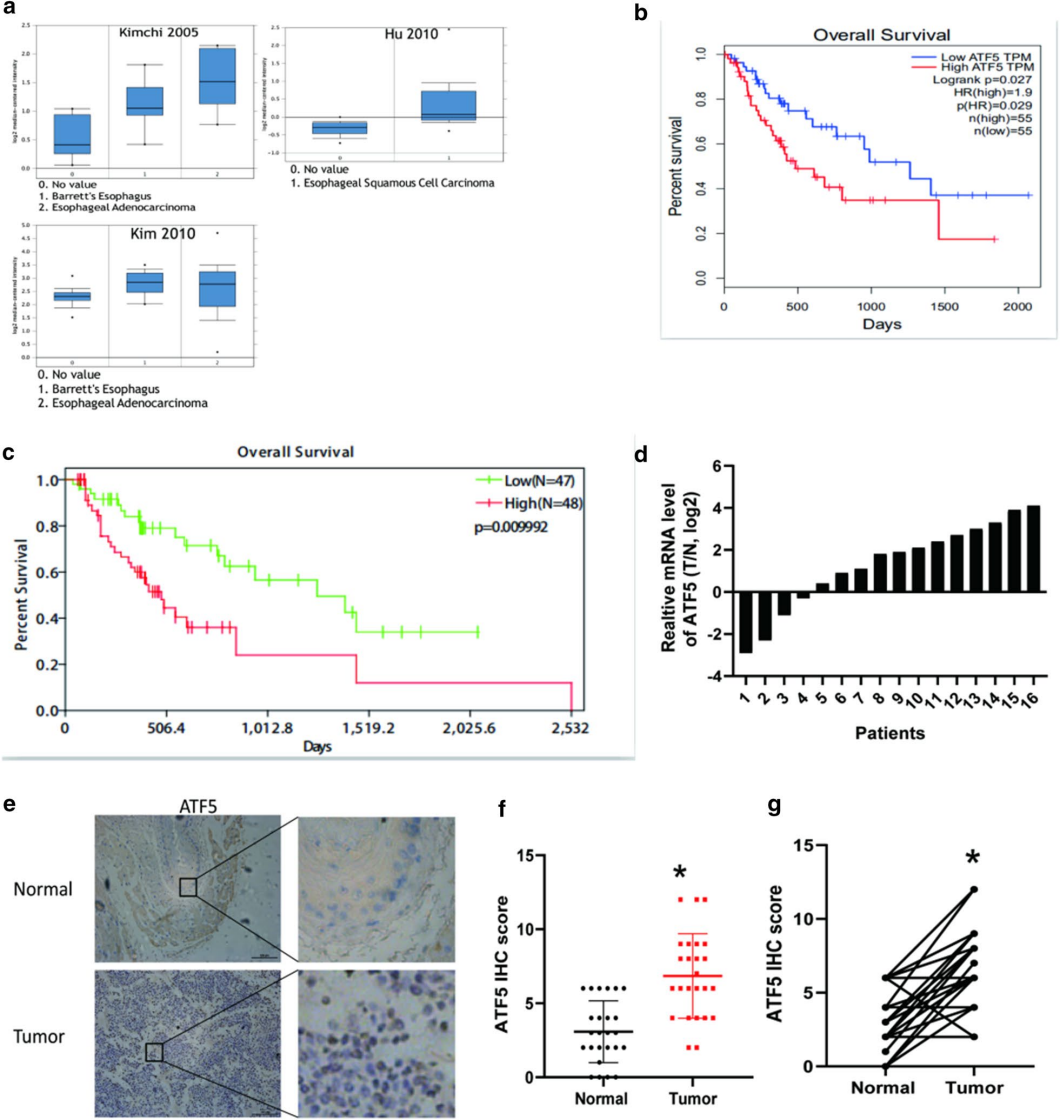
ATF5 is upregulated in ESCA. **a** ATF5 expression level in normal and ESCA tissues via the Oncomine database. **b**–**c** Kaplan–Meier plot of the overall survival via the GEPIA database (**b**) and TCGA database (**c**) Patients with ESCA were classified by ATF5 expression level. **d** mRNA level of ATF5 in 16 paired ESCA tissues. **e**–**g** Analysis of ATF5 expression level in paired ESCA tissues via IHC; Scale bar 100 μm; *p < 0.01 (**f**) and *p < 0.01 (**g**)

We searched the Oncomine database to investigate the function of ATF5 in ESCA. According to the results, ATF5 was upregulated in both esophageal adenocarcinoma and esophageal squamous carcinoma (Fig. 1a). Subsequently, we searched the GEPIA database to determine the prognosis of patients with ATF5 overexpression. Patients with a high level of ATF5 expression had a shorter overall survival time than those with low expression level (Fig. 1b). Further, we analyzed the data of ESCA patients in the TCGA database and observed that the level of ATF5 expression was negatively correlated with the prognosis of patients (Fig. 1c). Cox regression analysis showed that ATF5 expression level was a risk factor for poor ESCA prognosis (Table 1 and Additional file 1: Table S3). Furthermore, data from Human Protein Atlas revealed that patients with higher ATF5 expression in renal cancer and endometrial cancer have lower overall survival time (Additional file 2: Figure S1A-B). Then, we detected the ATF5 level in 16-paired ESCA and normal tissues from Tongji hospital. RT-PCR assay showed that the ATF5 mRNA level was much higher in ESCA tissues than in matched normal tissues (Fig. 1d). IHC analysis showed that the level of ATF5 protein was higher in ESCA tissues than in matched normal tissues (Fig. 1e–g).

**Table 1:** Cox regression analysis from TCGA database

Variates	*p*	HR	HR_95% CI	
			Lower	upper
ATF5	0.037	1.346	1.017	1.780
Gender	0.049	0.238	0.057	0.991
N	0.002	1.639	1.196	2.247
Age	0.172	–	–	–
Histological grade	0.232	–	–	–
M	0.064	–	–	–
T	0.075	–	–	–

### ATF5 overexpression promotes the proliferation, migration, and invasion capacity of ESCA cells

**Fig. 2:**
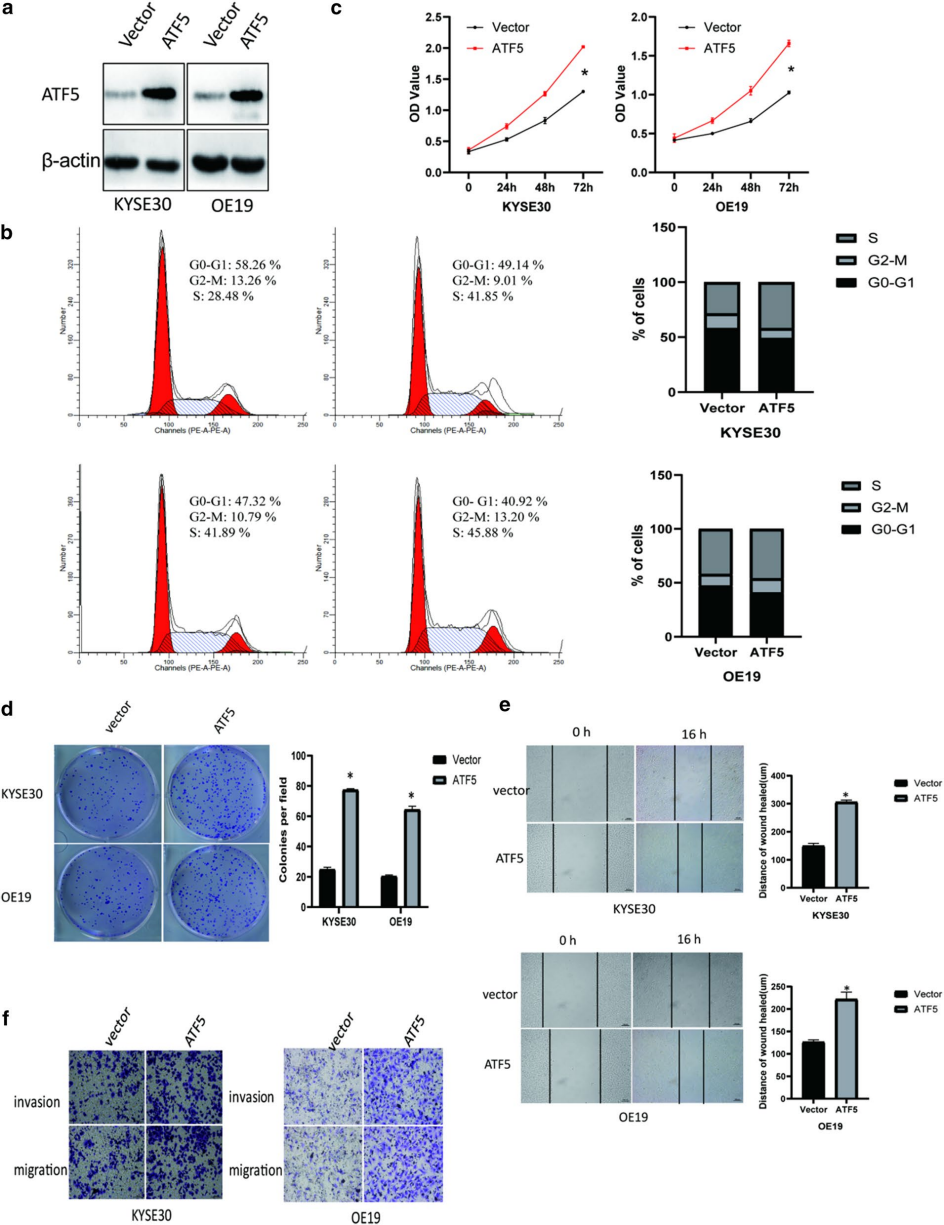
ATF5 overexpression promotes the proliferation, migration, and invasion ability of ESCA cells. **a** Analysis of ATF5 expression level in vector versus ATF5 cells by western blot. **b** Analysis of cell cycle in vector versus ATF5 cells by PI assay. **c** Analysis of cell proliferation ability in vector versus ATF5 cells by CCK8 assay; *p < 0.01. **d** Analysis of tumorigenicity in vector versus ATF5 cells by colony formation assay; *p < 0.01. **e** The migration ability of identified cells stably expressing the empty vector or ATF5 was analyzed through a wound healing assay; Scale bar 100 μm; *p* < 0.01. **f** Analysis of invasion and migration ability in vector versus ATF5 cells by Transwell assay; Scale bar 100 μm (left) or 50 μm (right)

We transfected KYSE30 and OE19 cells with pcDNA-3.1(+) (empty vector) and pcDNA-3.1(+)-ATF5 plasmid (Fig. 2a) to further investigate the biological function of ATF5 in ESCA. In addition, we conducted PI and CCK8 assays to determine whether ATF5 overexpression could promote cell proliferation and cell cycle progression. Based on PI analysis, ATF5 overexpression decreased the G0–G1 phase ratio but increased S phase ratio (Fig. 2b). Consistent with PI analysis, CCK8 assay revealed that ATF5 cells had higher proliferation ability than vector cells (Fig. 2c). Also, colony formation assay showed that ATF5 overexpression increased the size and number of cell colony (Fig. 2d).

Based on the above results, we hypothesized that ATF5 might promote the metastasis of ESCA cells. Thus, we conducted Transwell assays which revealed that ATF5 overexpression increased the number of cells, indicating that ATF5 enhanced the ability of cells to migrate and invade Matrigel (Fig. 2e).This property of ATF5 was also confirmed by wound healing assays (Fig. 2f).

### Silencing of ATF5 inhibits the proliferation, migration, and invasion ability of ESCA cells

**Fig. 3:**
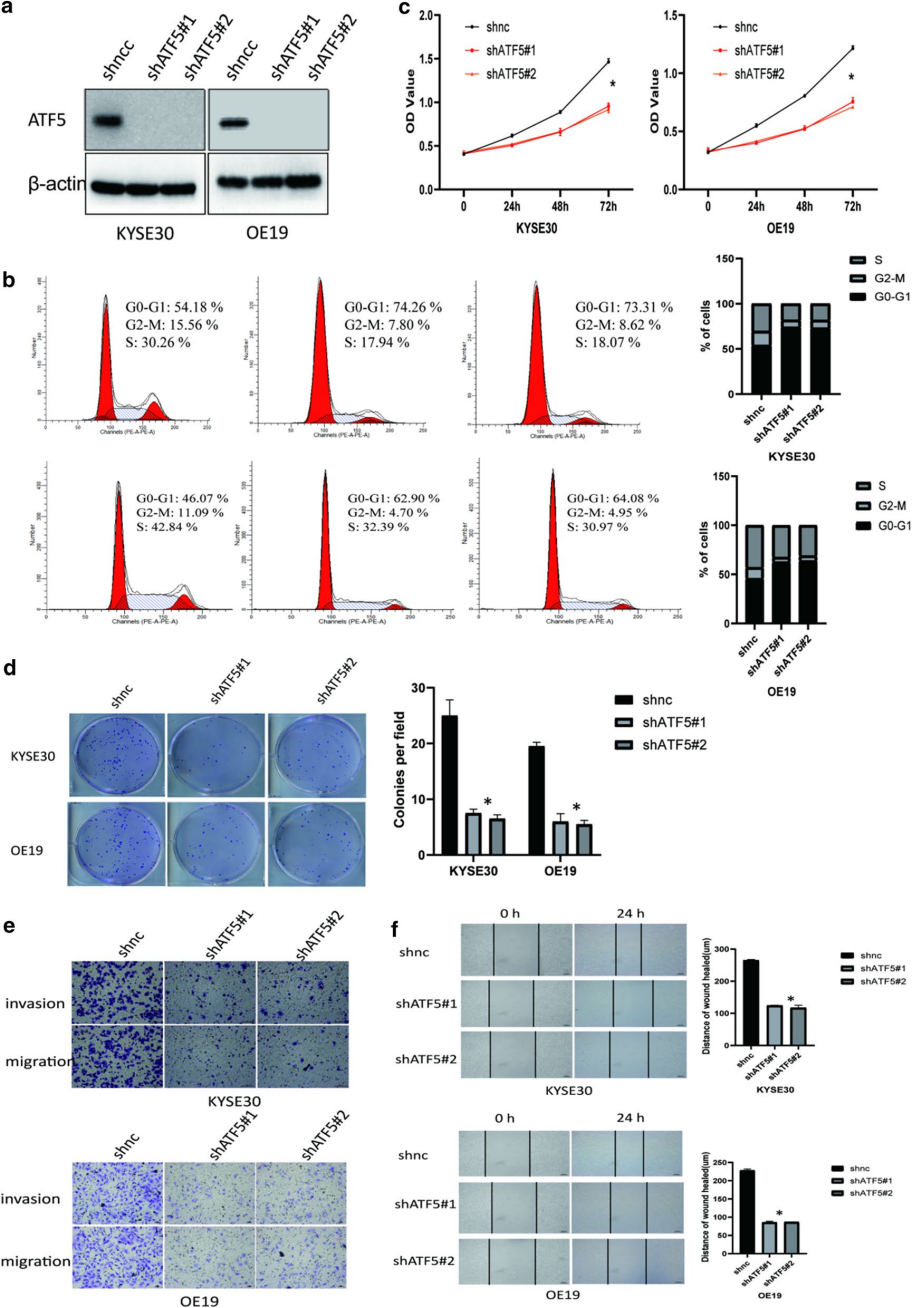
Silencing of ATF5 inhibits the proliferation, migration, and invasion ability of ESCA cells. **a** Analysis of ATF5 expression level in shnc versus shATF5 cells by western blot. **b** Analysis of cell cycle in shnc versus shATF5 cells by PI assay. **c** Analysis of cell proliferation ability in shnc versus shATF5 cell by CCK8 assay; *p < 0.01. **d** Analysis of tumorigenicity in shnc versus shATF5 cells by colony formation assay; *p < 0.01. **e** Analysis of invasion and migration ability of shnc versus shATF5 cells by Transwell assay; Scale bar 100 μm(up) or 50 μm(down). **f** The migration ability of identified cells stably expressing shnc and shATF5 was analyzed through a wound healing assay; Scale bar 100 μm;* p* < 0.01

To further validate the biological function of ATF5 in cell proliferation, invasion, and migration, we stably knocked down ATF5 in KYSE30 and OE19 cell lines using two different lentivirus-mediated shRNAs (Fig. 3a). The results from PI and CCK8 assays showed that ATF5 silencing inhibited the proliferation of the cells (Fig. 3b, c). Colony formation assay indicated that ATF5 knock-down suppressed the tumorigenicity of ESCA cells (Fig. 3d). Also, the Transwell assay showed that ATF5 silencing inhibited the migration and invasion ability of ESCA cells (Fig. 3e). The observation was further confirmed by the wound healing assay (Fig. 3f).

### The novel role of ATF5 in the HIF1 signaling pathway

**Fig. 4:**
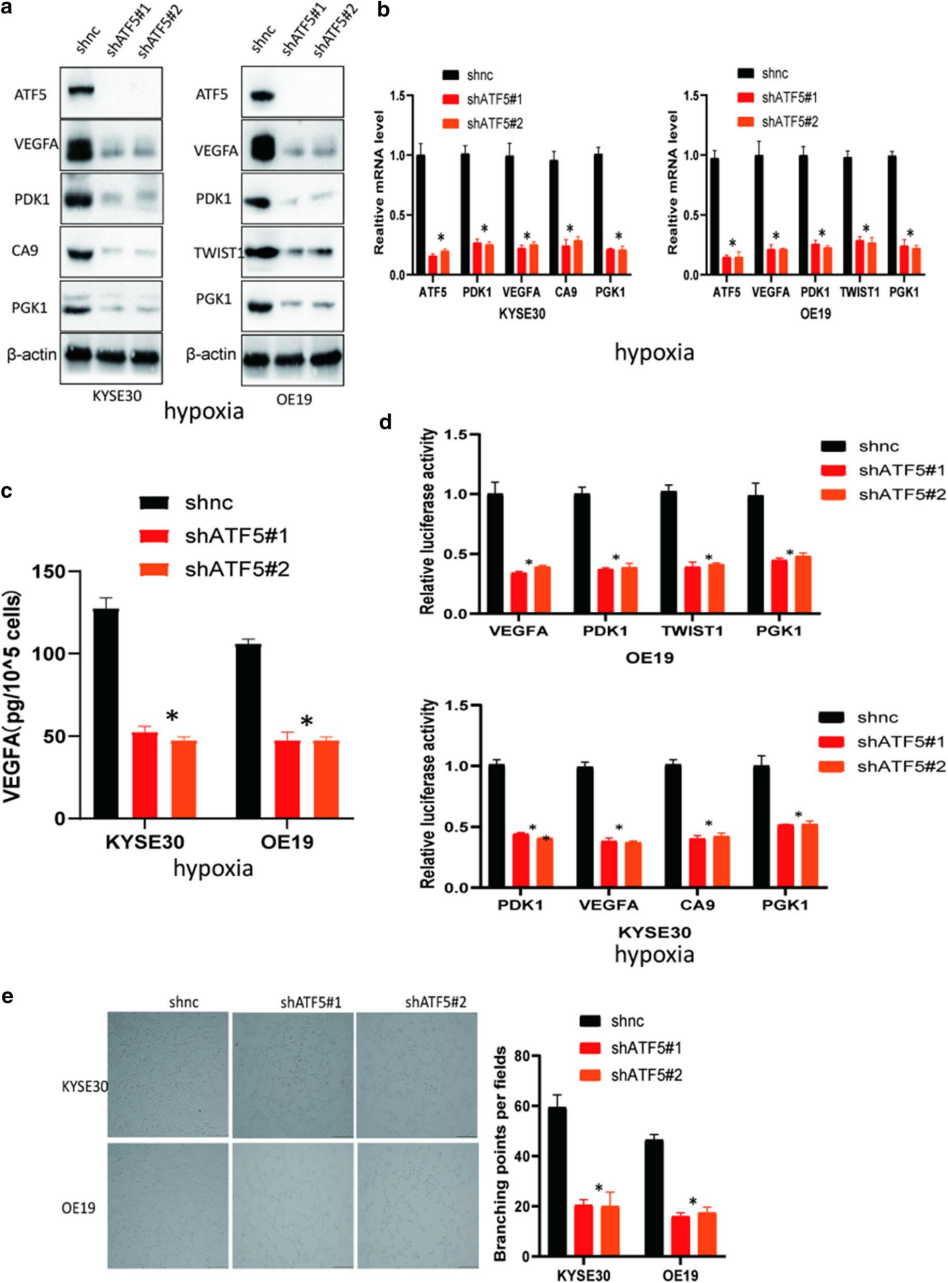
The novel role of ATF5 in the HIF1 signaling pathway. **a** Detection of the expression level of identified proteins in shATF5 versus shnc cells by western blot in hypoxia. **b** Detection of the expression level of identified mRNAs in shATF5 versus shnc cells by RT-PCR; *p* < 0.01 in hypoxia. **c** Evaluating the secretion of VEGFA in the identified cells by ELISA; *p* < 0.01 in hypoxia. **d** Dual-luciferase assays were performed to detect the luciferase activation of identified genes in ESCA cells transfected with shnc and shKDM4C; *p* < 0.01 in hypoxia. **e** Investigating the tube formation ability of HUVECs induced by supernatant from medium of identified cells in hypoxia; Scale bar 100 μm; *p* < 0.01

To further investigate the mechanism underlying the ATF5 functions, we conducted an RNA sequencing (RNA-seq) analysis to identify the downstream genes using HEK 293T cells stably expressing shnc and shATF5. Interestingly, six proteins (PDK1, CA9, PAI1, VEGFA, TWIST1, PGK1), belonging to the HIF1 signaling pathway, were downregulated in the shATF5 group and could be transcriptionally activated by HIF1 transcriptional complex. The heat map indicated the six differentially expressed genes in the shnc group and shATF5 groups (Additional file 2: Figure S1C and Additional file 1: Table S4). To further validate the function of ATF5 in the HIF1 signaling pathway, we examined the expression of the downstream genes of HIF1 transcriptional complex in shnc versus shATF5 ESCA cells. Western blot analysis showed that shATF5 cells exhibited a lower expression level of identified proteins compared with shnc cells in hypoxia (Fig. 4a). Besides, the expression of ATF5 had no visual change in cells treated by hypoxia compared with that in cells treated by normoxia (Additional file 2: Figure S1D). RT-PCR analysis further revealed that ATF5 silencing reduced the mRNAs expression of the identified proteins in hypoxia (Fig. 4b). Moreover, Results from dual-luciferase assays showed that loss of ATF5 dramatically decreased the luciferase activation of identified genes in ESCA cells in hypoxia (Fig. 4c). Next, we analyzed the concentration of VEGFA in the supernatant of cultured ESCA cell lines using ELISA. According to the results, silencing of ATF5 significantly inhibited VEGFA secretion in the ESCA cells in hypoxia (Fig. 4d). To further validate this, we conducted tube formation assay using HUVECs. Consistently, the supernatant from shATF5 cells significantly suppressed the tube formation ability of HUVECs compared with that from shnc cells in hypoxia (Fig. 4e). The above findings could be also observed in normoxia (Additional file 2: Figure S2).

### ATF5 functions as a novel coactivator in HIF1 transcription complex by binding to HIF1α

**Fig. 5:**
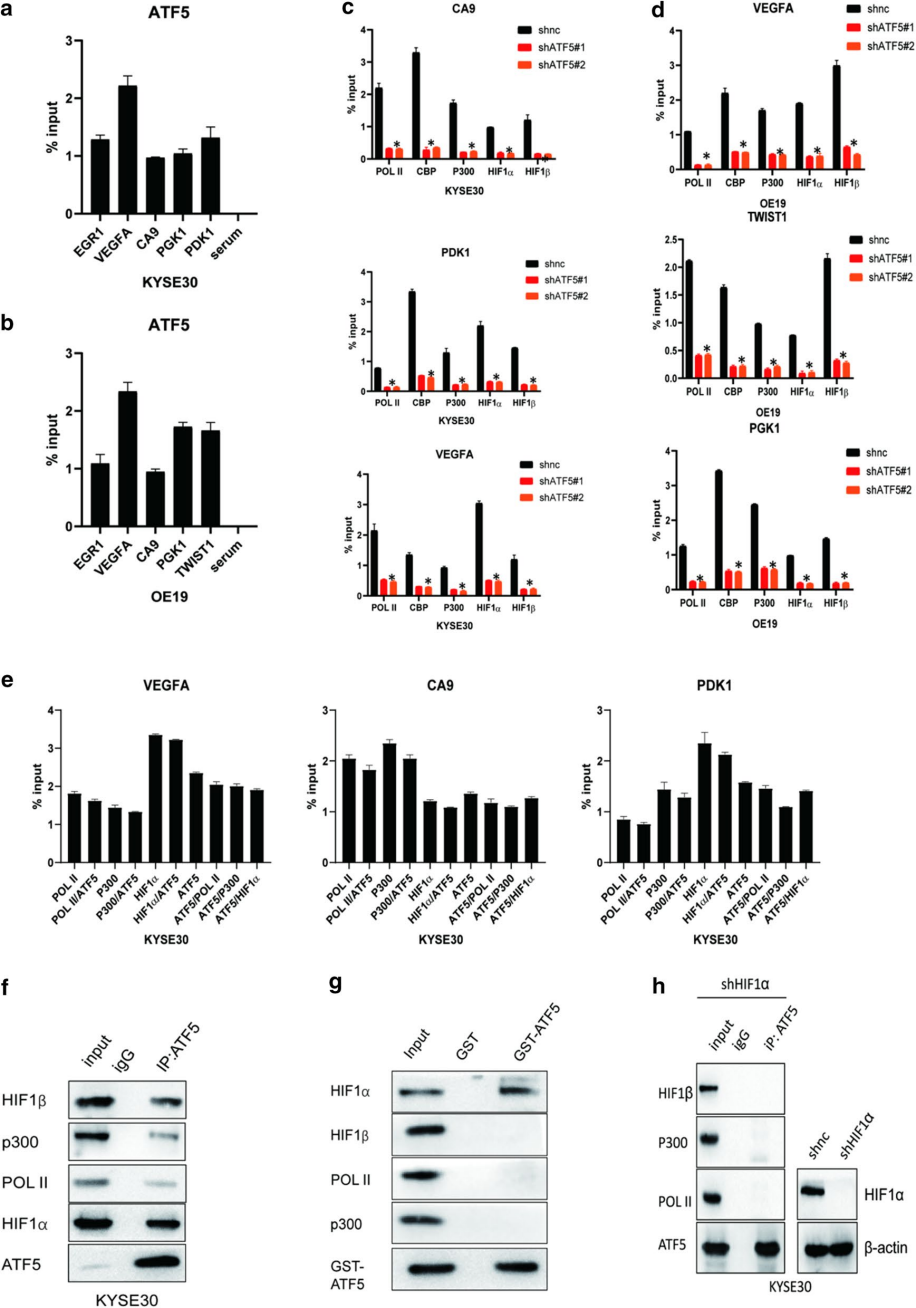
ATF5 acts as a novel coactivator in the HIF1 transcription complex by binding to HIF1α. **a**, **b** Examining the interaction between ATF5 and HIF1 target gene promoters by CHIP in hypoxia. **c**, **d** Investigating the interaction between HIF1 transcriptional complex and VEGFA promoter in shnc and shATF5 cells by CHIP in hypoxia; *p* < 0.01. **e** Investigating the interaction between ATF5 and HIF1 transcriptional complex on endogenous VEGFA promoters by ChIP/Re-ChIP in hypoxia. **f** Whole-cell extracts of KYSE30 cells were collected for IP analysis using the indicated antibodies, followed by IB analysis in hypoxia. **g** Investigating which protein of HIF1 transcriptional complex directly binding to ATF5 by GST pull down. **h** Whole-cell extracts of shATF5 KYSE30 cells were collected for IP analysis using the indicated antibodies, followed by IB analysis in hypoxia

Given that ATF5 promotes the expression of HIF1 target genes, we aimed to investigate the detailed molecular mechanism. As such, we hypothesized that ATF5 might directly bind to the promoters of HIF1 target genes. Thus, we carried out ChIP assays to investigate the binding of ATF5 to HIF1 target genes in 24 h hypoxia-treated cells. We selected the EGR1 promoter as a positive control [7]. Expectedly, ATF5 could bind to the promoters of HIF1 target genes (Fig. 5a, b). Next, we conducted an experiment to determine whether ATF5 was necessary for HIF1 transcriptional complex binding to HIF1 target genes. We first knocked-down the ATF5 gene in ESCA cell lines and performed ChIP assays to examine the interaction between HIF1 transcriptional complex and HIF1 target gene promoters. Silencing of ATF5 reduced the binding between HIF1 transcriptional complex and HIF1 target gene promoters in hypoxia (Fig. 5c, d). Next, we performed an experiment using ChIP/Re-ChIP to test whether there is a physical interaction between ATF5 and HIF1 transcriptional complex on HIF1 target gene promoters in 24 h hypoxia-treated KYSE30 cells. Interestingly, qPCR for PDK1, CA9, and VEGF promoters in precipitated DNA revealed Pol II bound to the same promoter with ATF5, so do p300 with ATF5 and HIF1α with ATF5. These results were further verified using the reciprocal ChIP/ReChIP of ATF5 with Pol II, ATF5 with p300 and ATF5 with HIF 1α in hypoxia (Fig. 5e). To further investigate the detailed molecular mechanism underlying the interaction between ATF5 and HIF1 transcriptional complex, we performed a co-IP analysis to establish whether there is interaction between ATF5 and HIF1 complex in hypoxia (Fig. 5f). Further, GST pull-down assays were carried out by incubating GST-ATF5 with in vitro-transcribed/translated HIF1α, HIF1β, P300 and Pol II. GST pull-down assay revealed that HIF1α directly bound GST-ATF5 in vitro but not HIF1β, P300 or Pol II (Fig. 5g). Silencing of HIF1α dramatically decrease the physical interaction between ATF5 and HIF1β, P300 or Pol II in hypoxia (Fig. 5h) and loss of ATF5 had no visual effect in HIF1α expression and stabilization in normoxia (Additional file 2: Figure S1E-F). Findings showed in Fig. 5a–f, h could be also observed in normoxia (Additional file 2: Figure S3).

### Inhibition ATF5 phenocopies HIF1α knockdown in tumorigenic properties in vitro

**Fig. 6:**
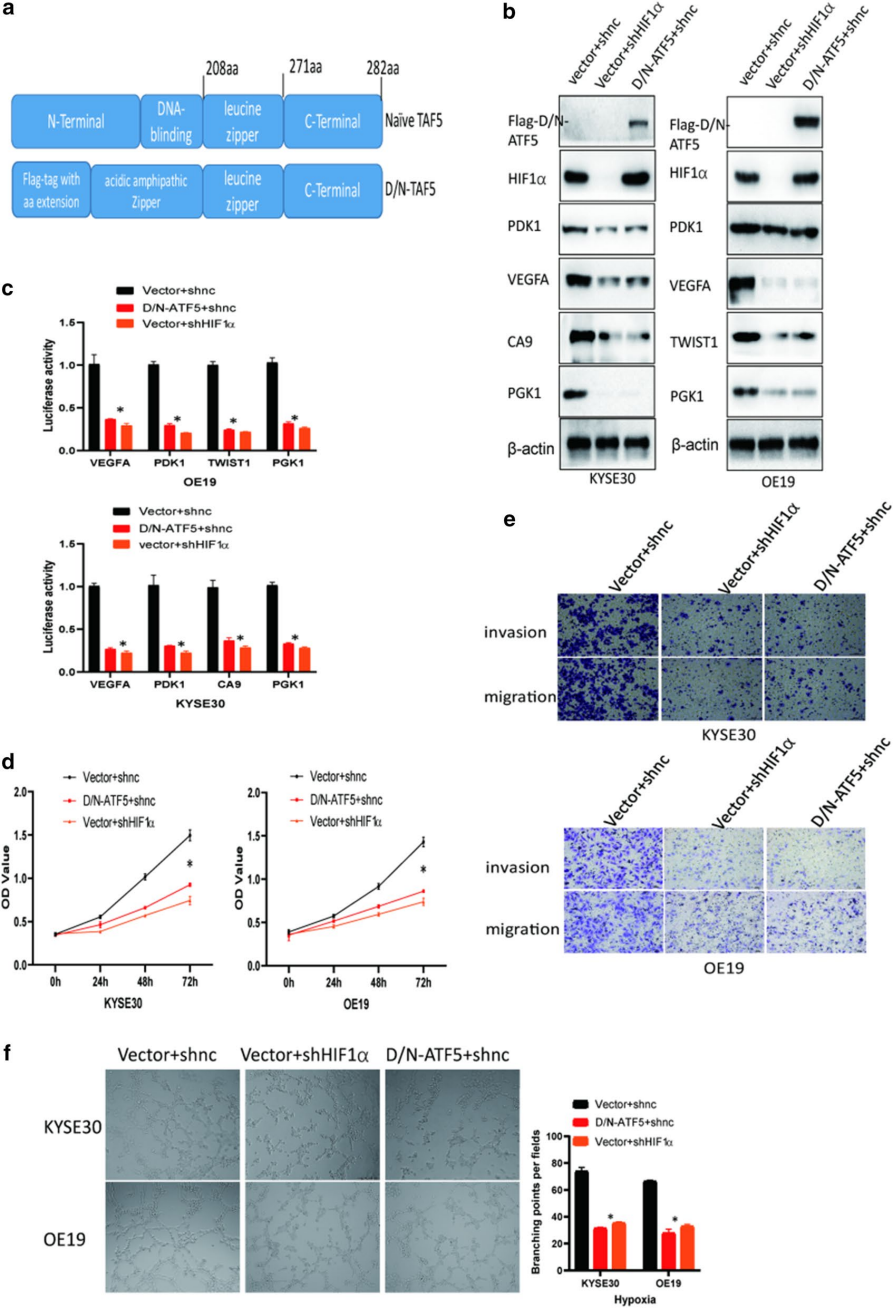
Inhibition ATF5 phenocopies HIF1α knockdown in tumorigenic properties in vitro. **a** The gene sequence of a dominant-negative ATF5 (D/N-ATF5). **b** Western blot showing the expression levels of the identified proteins in hypoxia. **c** Dual-luciferase assay showing the activation ability of identified gene promoters in identified cells in hypoxia; *p* < 0.01. **d** CCK8 assay showing the proliferation ability of identified cells in hypoxia; *p* < 0.01. **e** Transwell assay showing the invasion and migration ability of the identified cells in hypoxia; Scale bar 100 μm. **f** Tube formation ability of HUVECs induced by supernatant from medium of identified the cells in hypoxia; Scale bar 100 μm; *p* < 0.01

Next, we conducted a functional assay to examine whether ATF5 inhibition could phenocopy HIF1 inhibition. A dominant-negative ATF5 (D/N-ATF5) was designed as ATF5 antagonist (Fig. 6a) [21]. First, we constructed KYSE30 cells stably expressing vector + shnc, D/N-ATF5 + shnc, and vector + shHIF1α. Western blot assay indicated that the expression of the identified proteins was inhibited in cells expressing D/N-ATF5 and shHIF1α in hypoxia (Fig. 6b). In addition, dual-luciferase assay showed that cells expressing D/N-ATF5 and shHIF1α had similarly lower ability to activate the HIF1 target gene promoters compared with negative control cells in hypoxia (Fig. 6c). Furthermore, CCK8 and Transwell assays showed that cells expressing D/N-ATF5 and shHIF1α presented similarly lower proliferation, invasion, and migration ability relative to negative control cells in hypoxia (Fig. 6d, e). Consistently, tube formation assay showed that the medium from inhibition ATF5 and silencing HIF1α cells similarly inhibited tube formation ability of HUVECs in hypoxia (Fig. 6f). All above findings could be also observed in normoxia (Additional file 2: Figure S4).

### Silencing of ATF5 inhibited tumor angiogenesis and tumor proliferation in vivo

**Fig. 7:**
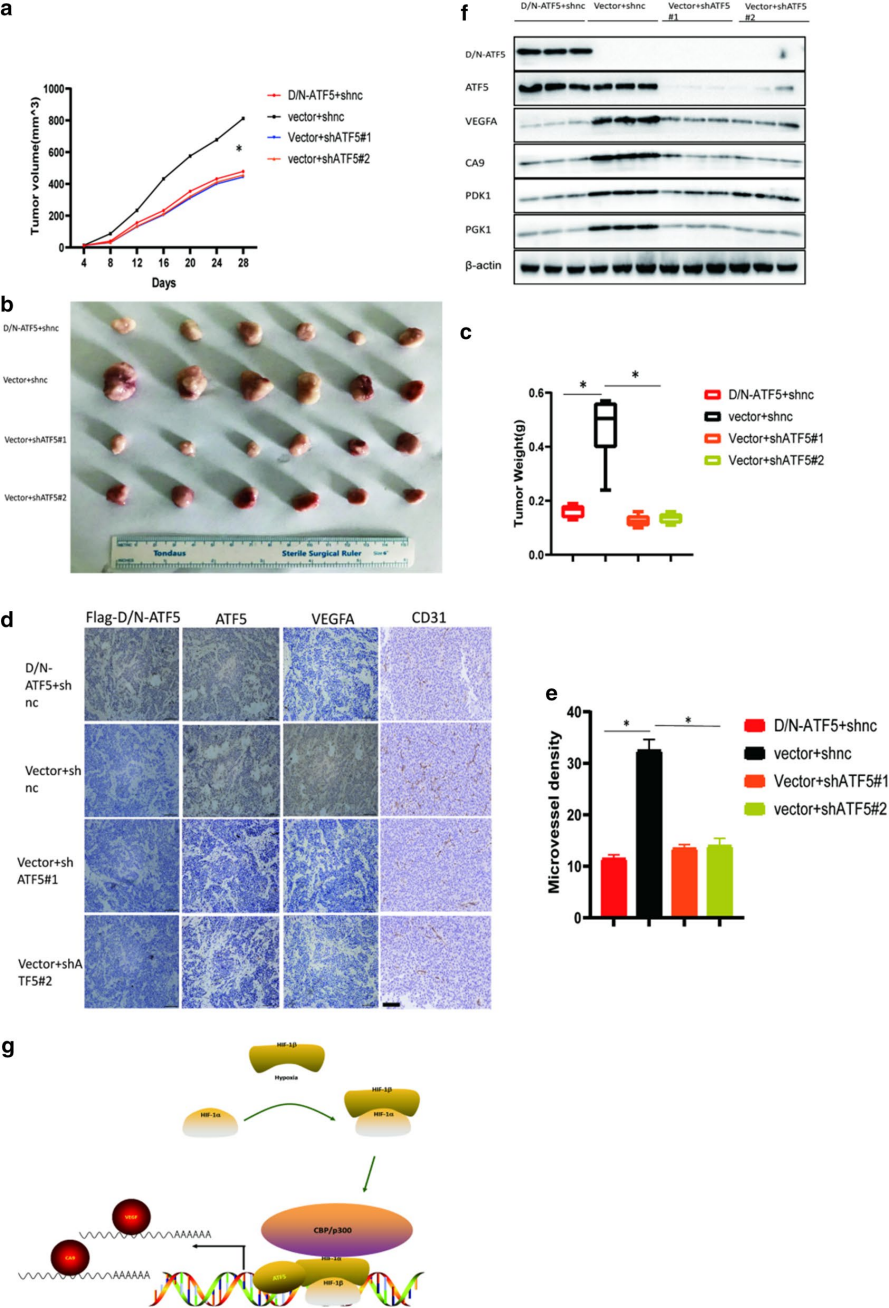
Silencing of ATF5 inhibited tumor angiogenesis and tumor proliferation in vivo. **a** The tumor growth curve among the four groups; *p* < 0.01. **b**, **c** Representative tumor images among the four groups (**b**), the right graph is showing the weight of the tumor (**c**); *p* < 0.01. **d**, **e**. Representative IHC staining of the identified protein among the four groups; the right graph showing the micro-vessel density of the tumors in the groups; Scale bar 100 μm. **f** Western blot assay showing the expression levels of the identified proteins in xenograft tumors. **g** Proposed model: ATF5/HIF1α interaction enhancing the binding of the HIF1 transcriptional complex on the promoters of the HIF1 target genes

ESCA xenograft mice model was conducted to investigate the role of ATF5 in tumorigenesis further. KYSE30 cells stably expressing vector + shnc, D/N-ATF5 + shnc, vector + shATF5#1, and vector + shATF5#2 were subcutaneously injected into nude mice (1 × 10^6^ cells per mouse). We calculated the volume of tumors every 4 days after a week of injections and plotted a tumor growth curve. Tumors volume of D/N-ATF5 and shATF5 groups grew significantly slower compared with the negative control group (Fig. 7a). After injecting the cells for 28 days, all mice were sacrificed to obtain the tumors which were then weighed and measured. As expected, tumor weight and volume from the D/N-ATF5 and shATF5 groups were significantly smaller than those from the negative control group (Fig. 7b, c). The expression of VEGFA and CD31 was investigated by IHC, and the results showed that VEGFA and CD31 was significantly lower in the D/N-ATF5 and shATF5 groups (Fig. 7d). Similarly, analysis of tumor micro-vessel density indicated that tumor micro-vessel was downregulated in D/N-ATF5 and shATF5 groups (Fig. 7e). Finally, we conducted a western blot assay to evaluate the expression levels of the HIF1 target genes. Based on the results, the expression of VEGFA, PDK1, CA9, and PGK1 was significantly reduced in the D/N-ATF5 and shATF5 groups (Fig. 7f).

## Discussion

Activating transcription factor 5 (ATF5) is well-known for its role in transcriptional activation of various genes, which in turn promotes the progression of various forms of cancer [23]. However, its precise role in the tumorigenesis of ESCA is still unclear. Herein, we confirmed that the expression level of ATF5 in ESCA patients is negatively related to their overall survival time. Also, we demonstrated that ATF5 promotes the proliferation, invasion, and migration of ESCA cells (Figs. 1, 2, 3).

Recent studies have linked ATF5 expression to the progression of various forms of cancer, but the mechanism underlying its role in tumorigenesis is largely unknown. HIF1 signaling pathway plays a crucial role in tumor formation and development, particularly tumor angiogenesis [6]. The pathways involved in HIF1α activation have been extensively studied. For example, HIF1α/HIF1β/CBP/P300, a conservative tetramer, can transcriptionally activate various target genes by binding to their promoters [26]; STAT3 acts as a co-activator of HIF1 transcriptional complex by binding to HIF1α [8]. In this study, we identified ATF5 as a novel co-activator of HIF1 transcriptional complex and demonstrated that ATF5 could be vital in the activation of HIF1 transcriptional complex in response to binding HIF1 target gene promoters. Besides, we confirmed that HIF1α acts as the link between ATF5 and HIF1 transcriptional complex (Fig. 5). However, a previous study revealed that P300 can directly acetylate ATF5, which is essential for Egr-1 gene expression and cell proliferation, as well as survival [20]. This difference might reflect the various biological characteristics of the different cell lines used in the present study. In addition, variations in experimental methods can also influence the results.

The potential of using ATF5 as a novel therapeutic target has been studied extensively. Herein, D/N-ATF5 was thought as an ATF5 antagonist [27]. Studies have shown that transfection of D/N-ATF5 transgene can trigger massive apoptosis in various cancer cell lines. Also, the use of D/N-ATF5 can significantly reduce the growth of various tumors in in vivo xenograft models by promoting apoptosis of cancer cells [17, 22, 23, 27, 28]. Here, we demonstrated a novel mechanism of D/N-ATF5 against tumor progression. D/N-ATF5 suppressed the activation of the HIF1 signaling pathway, and further inhibited tumor angiogenesis in *in-vivo* xenograft models (Figs. 6, 7).

In conclusion, this study reveals a novel mechanism underlying the activation of the HIF1 signaling pathway. Our studies demonstrate that ATF5 interacts with the HIF1 pathway. Our findings suggest that inhibition of ATF5 activity can be anti-tumorigenic and especially in certain HIF1-mediated tumorigenesis. These findings provide new insights into the molecular mechanism underlying ESCA progression and may be applied in the development of a novel therapeutic strategy for the treatment of esophageal cancer.

## Supplementary Information

**Additional file 1: Supplementary Table 1**. The RT-PCR primer sequences.** Supplementary Table 2**. The ChIP primers sequences.** Supplementary Table 3**. TCGA patients information.** Supplementary Table 4**. The differentially expressed genes.

**Additional file 2: Figure S1.** **a**, **b** Kaplan–Meier plot of overall survival via the Human Protein Atlas database: patients with renal cancer (**a**) and endometrial cancer (**b**) were classified by the ATF5 expression level. **c** Analysis of differentially expressed genes in shnc versus shATF5 cells by RNA sequencing. **d** Investigating the expression level of ATF5 in cells treated by normoxia or hypoxia. **e**, **f** Investigating the expression (**e**) and poly-ubiquitin (**f**) level of HIF1α in identified cells treated by normoxia. **Figure S2**. **a** Detection of the expression level of identified proteins in shATF5 vs. shnc cells by western blot in normoxia. **b** Detection of the expression level of identified mRNAs in shATF5 versus shnc cells by RT-PCR; *p* < 0.01 in normoxia. **c** Evaluating the secretion of VEGFA in the identified cells by ELISA in normoxia; *p* < 0.01. **d** Dual-luciferase assays were performed to detect the luciferase activation of identified genes in ESCA cells transfected with shnc and shKDM4C in normoxia; *p* < 0.01. **e** Investigating the tube formation ability of HUVECs induced by supernatant from medium of identified cells in normoxia; Scale bar 100 μm; *p* < 0.01. **Fgure S3** **a**, **b** Examining the interaction between ATF5 and HIF1 target gene promoters by CHIP in normoxia. **c**, **d** Investigating the interaction between HIF1 transcriptional complex and VEGFA promoter in shnc and shATF5 cells by CHIP in normoxia; *p* < 0.01. **e** Investigating the interaction between ATF5 and HIF1 transcriptional complex on endogenous VEGFA promoters by ChIP/Re-ChIP in normoxia. **f** Whole-cell extracts of KYSE30 cells were collected for IP analysis using the indicated antibodies, followed by IB analysis in normoxia. **g** Whole-cell extracts of shATF5 KYSE30 cells were collected for IP analysis using the indicated antibodies, followed by IB analysis in normoxia. **Figure S4** **a** Western blot showing the expression levels of the identified proteins in normoxia. **b** Dual-luciferase assay showing the activation ability of identified gene promoters in identified cells in normoxia; *p* < 0.01. **c** CCK8 assay showing the proliferation ability of identified cells in normoxia; *p* < 0.01. **d** Transwell assay showing the invasion and migration ability of the identified cells in normoxia; Scale bar 100 μm. **e** Tube formation ability of HUVECs induced by supernatant from medium of identified the cells in normoxia; Scale bar 100 μm; *p* < 0.01.

## Data Availability

All data generated or analyzed during this study are included in this published article.

## Abbreviations

ESCA: Esophageal cancer
HIF1: Hypoxia-inducible factor 1
ATF5: Activating transcription factor 5
VEGF: Vascular endothelial growth factor
IHC: Immunohistochemistry
ELISA: Enzyme-linked immunosorbent assay
ChIP: Chromatin immunoprecipitation
