# Supplementary material for: ATF5 and HIF1α cooperatively activate HIF1 signaling pathway in esophageal cancer
Source: Cell Commun Signal. 2021 May 12;19:53. doi: 10.1186/s12964-021-00734-x (PMC8117505; doi:10.1186/s12964-021-00734-x)
Supplement: Supplementary file 2 — Additional file 1: Supplementary Table 1. The RT-PCR primer sequences. Supplementary Table 2. The ChIP primers sequences. Supplementary Table 3. TCGA patients information. Supplementary Table 4. The differentially expressed genes. [file 12964_2021_734_MOESM2_ESM.docx]

| Supplementary table 1 | | | | | |
| --- | --- | --- | --- | --- | --- |
| GENE | forwards | | | rewards | |
| ATF5 | TGGCTCGTAGACTATGGGAAA | | | ATCAACTCGCTCAGTCATCCA | |
| VEGFA | AGGGCAGAATCATCACGAAGT | | | AGGGTCTCGATTGGATGGCA | |
| EGF | TGGATGTGCTTGATAAGCGG | | | ACCATGTCCTTTCCAGTGTGT | |
| PGK1 | TGGACGTTAAAGGGAAGCGG | | | GCTCATAAGGACTACCGACTTGG | |
| PDK1 | CTGTGATACGGATCAGAAACCG | | | TCCACCAAACAATAAAGAGTGCT | |
| CA9 | GGATCTACCTACTGTTGAGGCT | | | CATAGCGCCAATGACTCTGGT | |
|  | | | | | |
| Supplementary table 2 | | | | |  |
| GENE | | forwards | backwards | |  |
| EGR1 | | CTGAGCAGCCGCGCG | CTCGCTCCCTCCCTC | |  |
| PDK1 | | CTTCAAGTTGTCCCA | TGAGAACACGTGCC | |  |
| VEGFA | | AGCTCCACAAACTTGGT | TCATCTGGCCTGCAGACATC | |  |
| CA9 | | TTGGCTAGAG TATGAGGG | CTCCCCACTGCCTGCCA | |  |
| PGK1 | | CTTCTGAGAGGTCTCTA | GAGGCTGCCCTACCAG | |  |
| TWIST 1 | | GGGAAAGGAGGGCCC | GGCCAAACCGCGGCGGC | |  |

| TCGA patients information | | | | | | | | | |
| --- | --- | --- | --- | --- | --- | --- | --- | --- | --- |
| sampleID | age_at_initial_pathologic_diagnosis | gender | neoplasm_histologic_grade | pathologic_M | pathologic_N | pathologic_T | ATF5 | OS | OS.time |
| TCGA-LN-A49L-01 | 44 | MALE | G2 | M0 | N0 | T2 | 12.9801 | 1 | 318 |
| TCGA-V5-A7RC-06 | 55 | MALE | GX |  |  |  | 12.8407 | 1 | 104 |
| TCGA-LN-A4A8-01 | 52 | MALE | G2 | M0 | N0 | T2 | 12.2508 | 0 | 472 |
| TCGA-L5-A8NN-01 | 81 | MALE | GX | M0 | N1 | T3 | 11.8848 | 0 | 167 |
| TCGA-IG-A5S3-01 | 69 | FEMALE | G2 | M0 | N0 | T3 | 11.7462 | 0 | 712 |
| TCGA-VR-A8EZ-01 | 47 | MALE | G2 | M0 | N3 | T3 | 11.7189 | 1 | 553 |
| TCGA-2H-A9GG-01 | 66 | MALE | G2 | M0 | N1 | T3 | 11.489 | 1 | 610 |
| TCGA-VR-A8EP-01 | 51 | MALE | G3 | M0 | N2 | T3 | 11.3749 | 0 | 824 |
| TCGA-V5-A7RB-01 | 59 | MALE | GX | MX | N1 | T0 | 11.2493 | 1 | 161 |
| TCGA-IG-A4QT-01 | 56 | MALE | G2 | M0 | N0 | T3 | 10.9518 | 1 | 283 |
| TCGA-L7-A56G-01 | 65 | MALE | G3 |  |  |  | 10.8064 | 1 | 330 |
| TCGA-V5-A7RC-01 | 55 | MALE | GX |  |  |  | 10.669 | 1 | 104 |
| TCGA-IG-A7DP-01 | 50 | FEMALE | G2 | MX | NX | T4a | 10.6668 | 0 | 452 |
| TCGA-2H-A9GL-01 | 74 | MALE | G3 | M0 | N1 | T3 | 10.6227 | 1 | 180 |
| TCGA-Z6-A8JE-01 | 57 | MALE | G3 | M0 | N1 | T3 | 10.5778 | 0 | 64 |
| TCGA-Z6-AAPN-01 | 57 | MALE | G1 | M0 | N0 | T3 | 10.4625 | 0 | 81 |
| TCGA-LN-A9FO-01 | 42 | MALE | G2 | M0 | N0 | T2 | 10.4587 | 0 | 4 |
| TCGA-LN-A8I1-01 | 67 | FEMALE | G3 | M0 | N0 | T2 | 10.4505 | 0 | 401 |
| TCGA-LN-A49Y-01 | 77 | MALE | G2 | M0 | N0 | T3 | 10.4133 | 0 | 379 |
| TCGA-VR-A8EO-01 | 49 | MALE | G3 | M0 | N0 | T3 | 10.3918 | 0 | 785 |
| TCGA-V5-A7RE-01 | 45 | MALE | G2 | M0 | N0 | T1 | 10.3673 | 0 | 500 |
| TCGA-LN-A49W-01 | 73 | MALE | G3 | M0 | N1 | T3 | 10.2957 | 0 | 403 |
| TCGA-V5-AASV-01 | 67 | MALE | G3 | MX | N0 | T3 | 10.1841 | 0 | 467 |
| TCGA-L5-A88V-01 | 60 | MALE | GX | MX | N1 | T3 | 10.1217 | 0 | 79 |
| TCGA-LN-A49K-01 | 66 | MALE | G2 | M0 | N0 | T3 | 10.106 | 1 | 180 |
| TCGA-LN-A9FP-01 | 60 | FEMALE | G3 | M0 | N0 | T2 | 10.0817 | 0 | 366 |
| TCGA-L5-A4OM-01 | 54 | FEMALE | GX |  | N0 | T1 | 10.033 | 1 | 1458 |
| TCGA-L5-A43C-01 | 81 | MALE | GX |  |  |  | 9.9878 | 0 | 96 |
| TCGA-2H-A9GO-01 | 58 | MALE | G3 | M1a | N1 | T3 | 9.9645 | 1 | 494 |
| TCGA-LN-A7HZ-01 | 49 | MALE | G2 | M0 | N0 | T2 | 9.9606 | 0 | 401 |
| TCGA-VR-AA7I-01 | 70 | MALE | G1 | M0 | N0 | T4 | 9.9564 | 1 | 484 |
| TCGA-IG-A4QS-01 | 71 | MALE | G3 | M0 | N2 | T3 | 9.9361 | 1 | 118 |
| TCGA-L5-A891-01 | 51 | MALE | GX | M0 | N1 | T3 | 9.9243 | 0 | 114 |
| TCGA-2H-A9GM-01 | 53 | MALE | G2 | M0 | N1 | T1 | 9.8996 | 1 | 424 |
| TCGA-IG-A5B8-01 | 72 | MALE | G1 | M0 | N0 | T3 | 9.8925 | 1 | 24 |
| TCGA-LN-A49M-01 | 62 | MALE | G1 | M0 | N0 | T2 | 9.8832 | 0 | 385 |
| TCGA-L5-A4OO-01 | 75 | MALE | G2 | M0 | N3 | T3 | 9.8507 | 0 | 101 |
| TCGA-VR-A8EW-01 | 57 | MALE | G2 | M0 | N2 | T3 | 9.835 | 1 | 247 |
| TCGA-L5-A4OW-01 | 56 | FEMALE | GX | M0 | N1 | T2 | 9.7634 | 1 | 217 |
| TCGA-2H-A9GK-01 | 43 | MALE | G3 | M0 | N1 | T3 | 9.7453 | 1 | 232 |
| TCGA-L5-A8NI-01 | 79 | MALE | G3 | M0 | N1 | T3 | 9.7187 | 1 | 410 |
| TCGA-LN-A5U5-01 | 57 | MALE | G2 | M1 | N1 | T3 | 9.6905 | 1 | 136 |
| TCGA-R6-A8WG-01 | 60 | MALE | G3 |  |  |  | 9.6751 | 1 | 386 |
| TCGA-IG-A3Y9-01 | 72 | MALE | G2 | M0 | N0 | T4 | 9.6673 | 1 | 26 |
| TCGA-S8-A6BW-01 | 51 | MALE | G1 | MX | N0 | T2 | 9.6345 | 0 | 620 |
| TCGA-VR-A8EX-01 | 63 | MALE | G2 | M1a | N1 | T1 | 9.6208 | 1 | 855 |
| TCGA-IG-A97H-01 | 36 | MALE | G3 | M0 | NX | T3 | 9.5913 | 0 | 441 |
| TCGA-LN-A4A9-01 | 58 | MALE | G1 | M0 | N0 | T2 | 9.5846 | 1 | 351 |
| TCGA-LN-A4MR-01 | 57 | MALE | G2 | M0 | N0 | T2 | 8.7265 | 0 | 402 |
| TCGA-LN-A7HX-01 | 72 | MALE | G2 | M0 | N0 | T2 | 8.7189 | 0 | 372 |
| TCGA-L5-A4OJ-01 | 70 | FEMALE | G3 | M0 | N0 | T1 | 8.7065 | 0 | 639 |
| TCGA-LN-A49U-01 | 62 | MALE | G1 | M0 | N0 | T3 | 8.7065 | 0 | 467 |
| TCGA-VR-AA4G-01 | 51 | FEMALE | G2 | M0 | N2 | T2 | 8.7026 | 0 | 549 |
| TCGA-JY-A6FD-01 | 51 | FEMALE | G1 | M0 | N0 | T3 | 8.6838 | 0 | 2069 |
| TCGA-L5-A8NE-01 | 77 | MALE | GX | M0 | N1 | T2 | 8.6764 | 0 | 1688 |
| TCGA-L5-A43E-01 | 74 | MALE | GX | M0 | N0 | T1 | 8.6732 | 0 | 920 |
| TCGA-LN-A5U6-01 | 54 | MALE | G2 | M0 | N1 | T2 | 8.6697 | 0 | 375 |
| TCGA-LN-A49X-01 | 44 | MALE | G2 | M0 | N0 | T3 | 8.6409 | 0 | 384 |
| TCGA-L5-A4OS-01 | 86 | FEMALE | G3 | M0 | N1 | T2 | 8.633 | 0 | 1782 |
| TCGA-VR-A8ER-01 | 54 | MALE | GX | M0 | N1 | T4 | 8.5976 | 1 | 378 |
| TCGA-JY-A93F-01 | 58 | FEMALE | G1 | M0 | N0 | T2 | 8.5802 | 0 | 731 |
| TCGA-L5-A43I-01 | 77 | MALE | GX | MX | N1 | T3 | 8.5727 | 1 | 556 |
| TCGA-IG-A97I-01 | 58 | MALE | G2 | M0 | N0 | T2 | 8.5577 | 0 | 370 |
| TCGA-ZR-A9CJ-01 | 65 | MALE | G3 | MX | N3 | T3 | 8.5498 | 1 | 600 |
| TCGA-LN-A9FR-01 | 70 | MALE | G2 | M0 | N1 | T2 | 8.5447 | 0 | 373 |
| TCGA-L5-A8NF-01 | 57 | MALE | GX | M1a | N0 | T1 | 8.5392 | 1 | 81 |
| TCGA-L5-A8NL-01 | 56 | MALE | GX | M0 | N1 | T3 | 8.527 | 0 | 402 |
| TCGA-L5-A4OI-01 | 79 | MALE | G2 | MX | N3 | T3 | 8.5119 | 0 | 608 |
| TCGA-R6-A6XG-01 | 64 | MALE | G2 |  |  |  | 8.5087 | 0 | 1168 |
| TCGA-L5-A4OT-01 | 77 | MALE | GX | M1a | N1 | T3 | 8.489 | 1 | 149 |
| TCGA-IG-A6QS-01 | 54 | MALE | G1 | M0 | N1 | T2 | 8.4829 | 1 | 303 |
| TCGA-LN-A9FQ-01 | 62 | MALE | G3 | M0 | N0 | T3 | 8.454 | 0 | 391 |
| TCGA-L5-A43J-01 | 90 | MALE | G3 | MX | N0 | T3 | 8.4343 | 1 | 131 |
| TCGA-2H-A9GN-01 | 70 | MALE | G3 | M0 | N1 | T3 | 8.4064 | 1 | 272 |
| TCGA-VR-AA7D-01 | 58 | MALE | G2 | M0 | N3 | T3 | 8.4021 | 1 | 279 |
| TCGA-KH-A6WC-01 | 82 | MALE | GX | M0 | N0 | T1 | 8.3826 | 0 | 191 |
| TCGA-L5-A8NR-01 | 81 | FEMALE | GX | M0 | N1 | T3 | 8.3779 | 0 | 265 |
| TCGA-LN-A49N-01 | 50 | MALE | G2 | M0 | N1 | T2 | 8.3634 | 0 | 378 |
| TCGA-LN-A49V-01 | 49 | MALE | G3 | M0 | N0 | T3 | 8.362 | 0 | 383 |
| TCGA-JY-A6FG-01 | 50 | MALE | G2 | M0 | N1 | T3 | 8.3499 | 1 | 1263 |
| TCGA-IC-A6RE-01 | 59 | MALE | G2 | M0 | N1 | T1 | 8.349 | 0 | 234 |
| TCGA-L5-A4OP-01 | 67 | FEMALE | G2 | MX | N0 | T1 | 8.279 | 0 | 218 |
| TCGA-L5-A8NT-01 | 69 | MALE | GX | M0 | N0 | T3 | 8.2719 | 0 | 825 |
| TCGA-LN-A4A3-01 | 61 | MALE | G2 | M0 | N1 | T3 | 8.271 | 0 | 388 |
| TCGA-XP-A8T6-01 | 54 | MALE | G2 | M0 | N1 | T2 | 8.2634 | 1 | 763 |
| TCGA-LN-A4A6-01 | 65 | MALE | G2 | M0 | N0 | T2 | 8.2503 | 0 | 391 |
| TCGA-VR-A8Q7-01 | 60 | MALE | G3 | M0 | N1 | T3 | 8.246 | 0 | 1590 |
| TCGA-JY-A93E-01 | 61 | MALE | G3 | M0 | N1 | T3 | 8.2457 | 0 | 767 |
| TCGA-2H-A9GH-01 | 44 | MALE | G2 | M0 | N1 | T1 | 8.2294 | 1 | 951 |
| TCGA-R6-A8WC-01 | 56 | MALE | G2 |  |  |  | 8.2092 | 0 | 70 |
| TCGA-LN-A5U7-01 | 46 | MALE | G1 | M0 | N0 | T2 | 8.0966 | 0 | 768 |
| TCGA-JY-A6FH-01 | 53 | MALE | G2 | M0 | N1 | T2 | 8.0558 | 0 | 1441 |
| TCGA-VR-AA4D-01 | 53 | MALE | G2 | M0 | N1 | T1 | 7.9546 | 1 | 1405 |
| TCGA-XP-A8T8-01 | 49 | MALE | G1 | M0 | N1 | T1 | 7.6396 | 0 | 437 |
| TCGA-VR-A8EY-01 | 44 | FEMALE | G1 | M0 | N0 | T3 | 7.3394 | 0 | 1025 |

| supplementary table 3 | | | | | | | | | |
| --- | --- | --- | --- | --- | --- | --- | --- | --- | --- |
| GeneID | shnc#1 | shnc#2 | shnc#3 | shATF5#1 | shATF5#2 | shATF5#3 | P_value | Description | **Accession** |
| VEGFA | 4.13885 | 5.38225 | 2.753477 | 0.715043 | 1.442513 | 1.62766 | 0.0249 | vascular endothelial growth factor A | NM_001025366 |
| PDK1 | 0.619713 | 0.69739 | 0.732179 | 0.133243 | 0.148041 | 0.250325 | 0.0026 | pyruvate dehydrogenase kinase 1 | NM_002610 |
| PAI1 | 1.85479 | 1.819504 | 1.786494 | 0.467705 | 0.486024 | 0.292159 | <0.0001 | serpin family E member 1 | NM_000602 |
| PGK1 | 13.98007 | 13.38406 | 12.68001 | 8.807717 | 8.257173 | 8.868371 | 0.0004 | phosphoglycerate kinase 1 | NM_000291 |
| CA9 | 0.87008 | 0.958304 | 0.765966 | 0.361576 | 0.35939 | 0.47329 | 0.0022 | carbonic anhydrase 9 | NM_001216 |
| TWIST1 | 6.432561 | 7.236196 | 7.680714 | 3.611866 | 3.327172 | 3.081564 | 0.0007 | twist family bHLH transcription factor 1 | NM_000474 |
