# Supplementary figures and images for: ATF5 and HIF1α cooperatively activate HIF1 signaling pathway in esophageal cancer
Source: Cell Commun Signal. 2021 May 12;19:53. doi: 10.1186/s12964-021-00734-x (PMC8117505; doi:10.1186/s12964-021-00734-x)

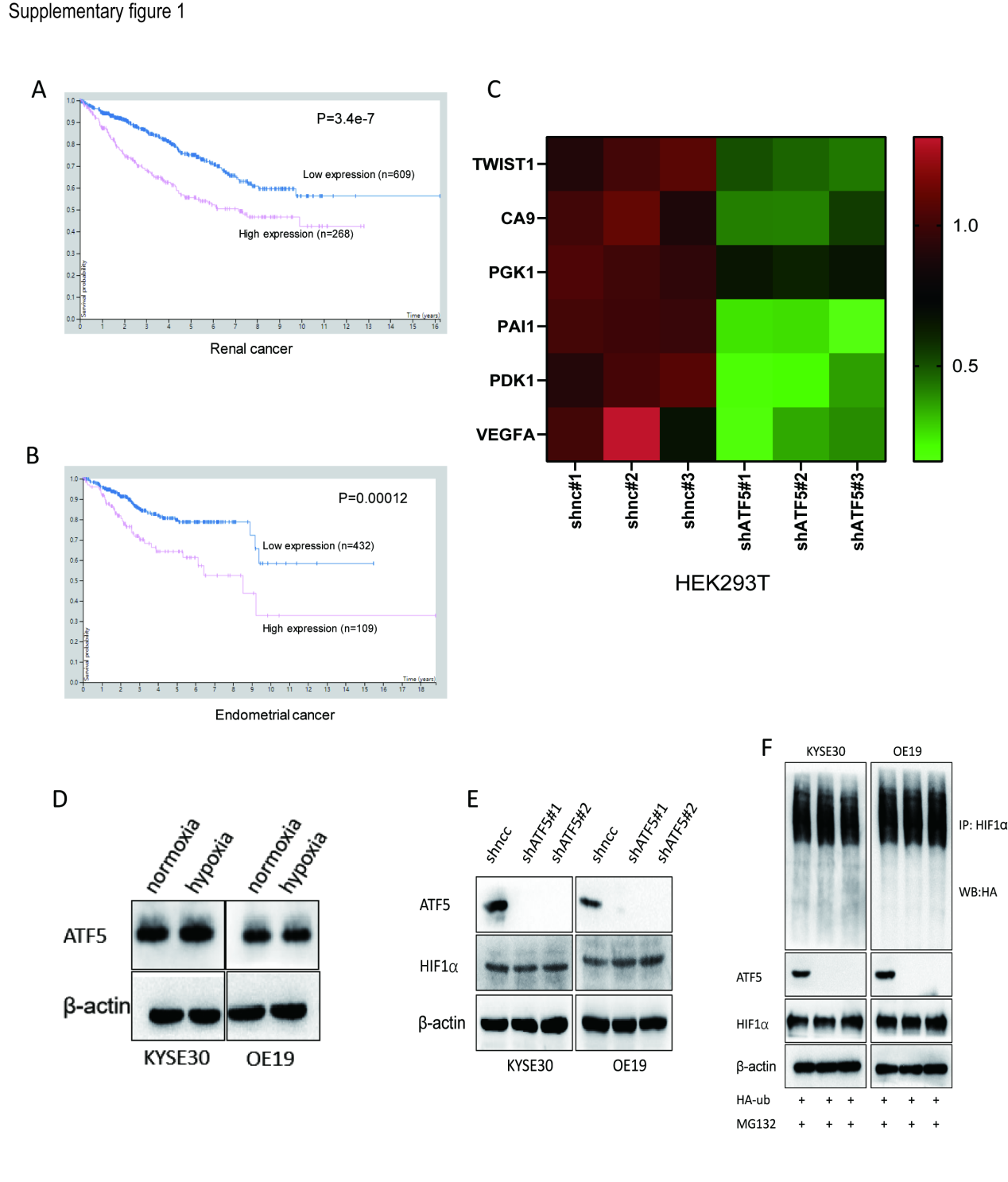

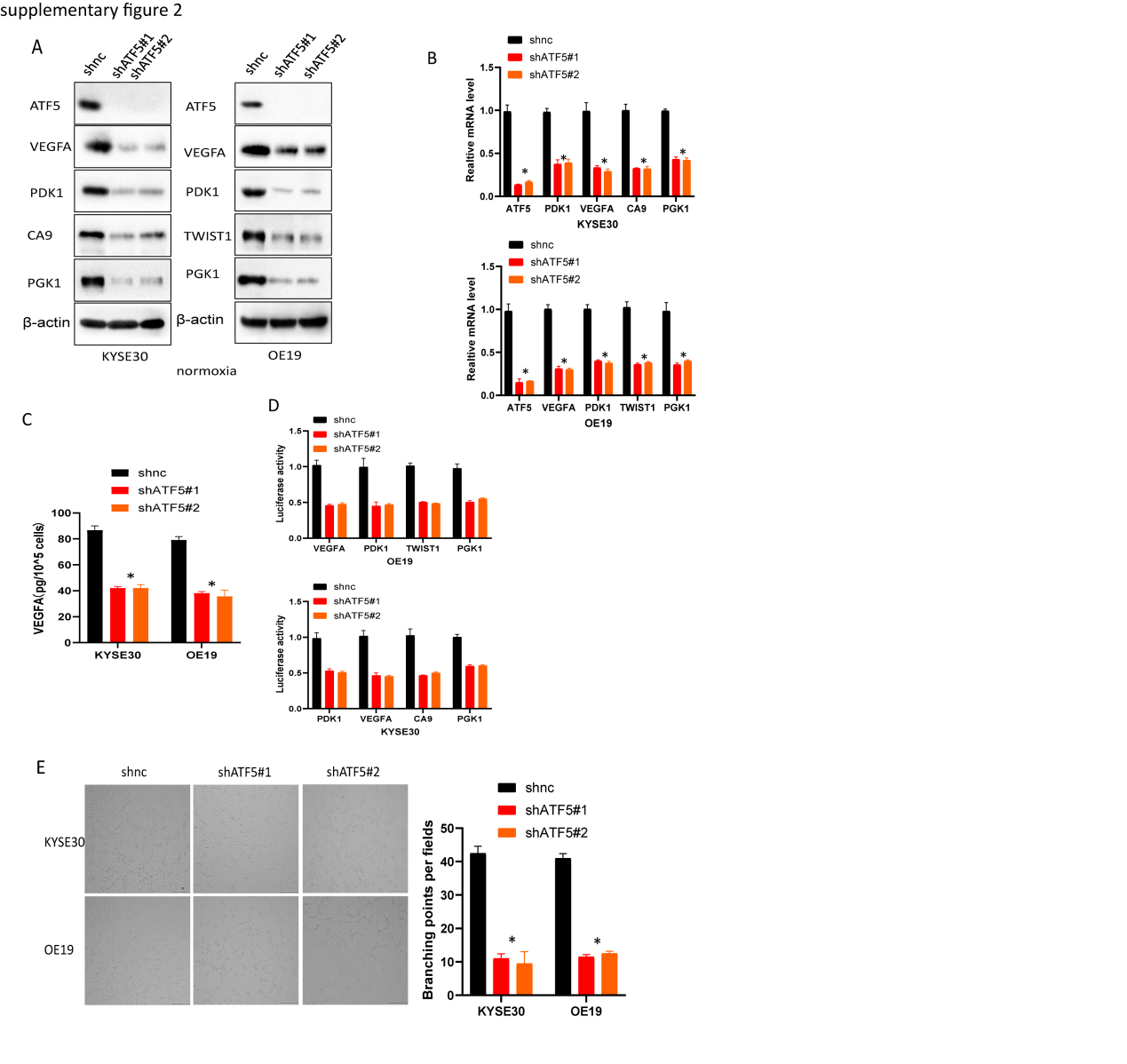

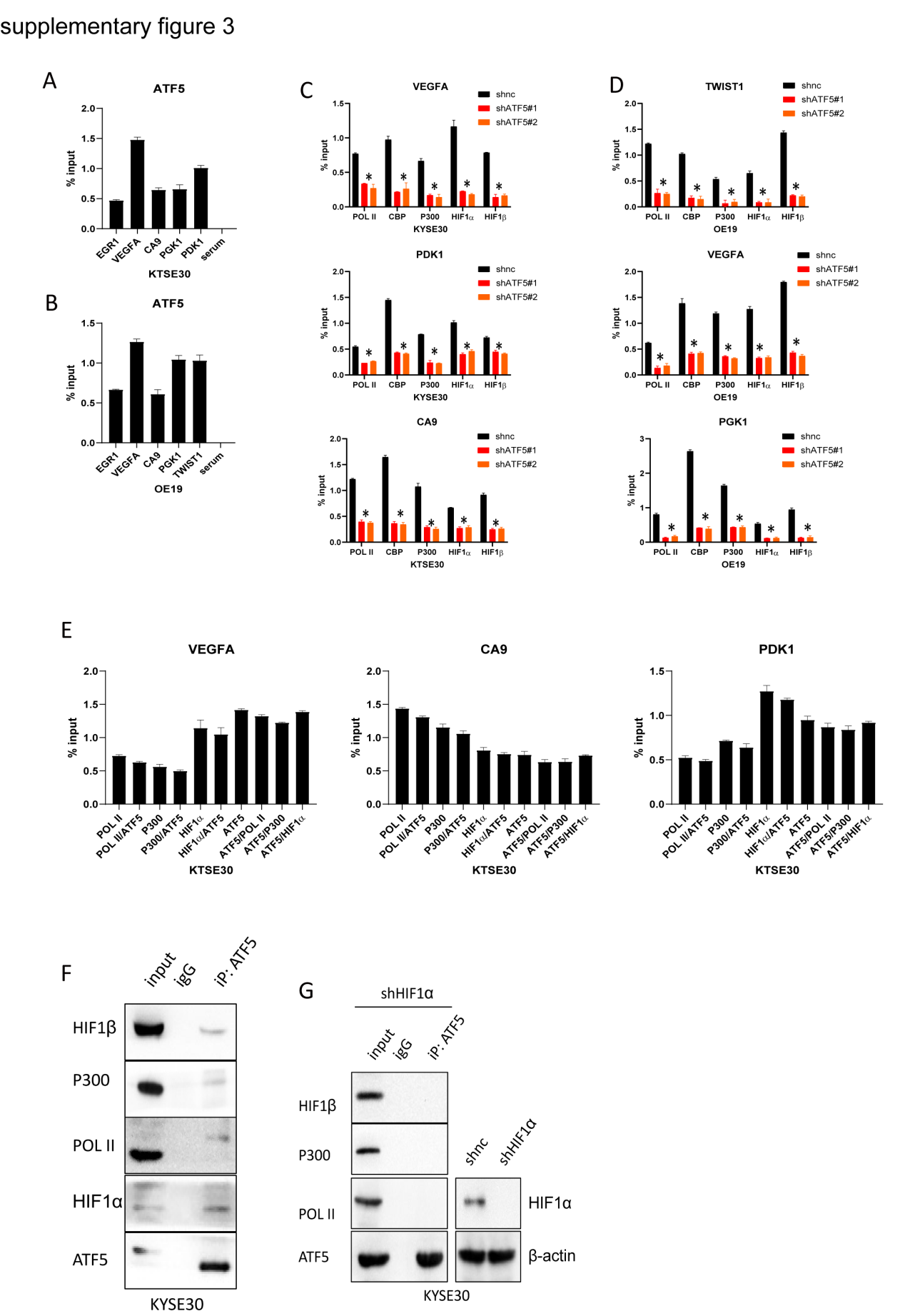

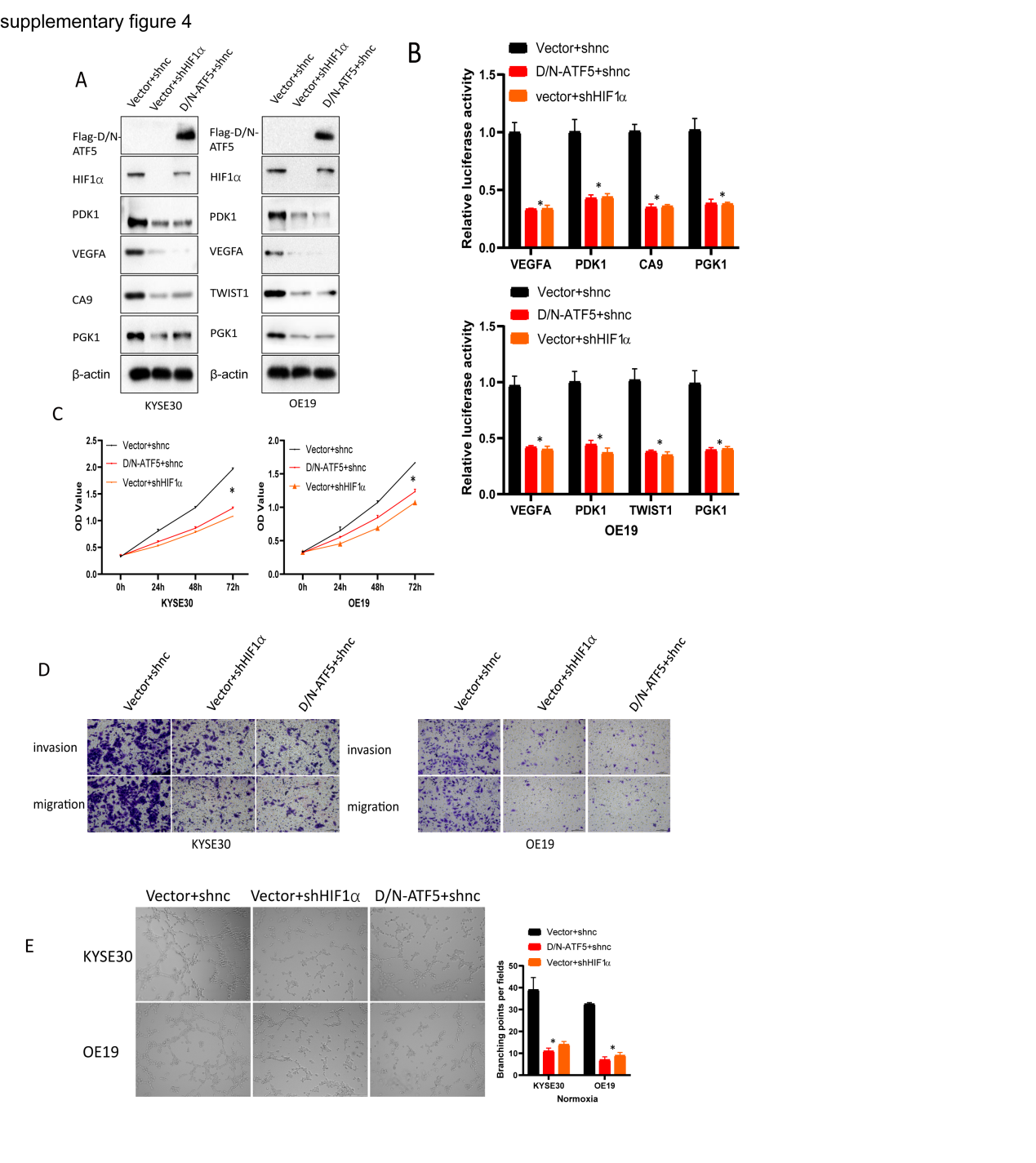

Supplement: Supplementary file 3 — Additional file 2: Figure S1. a, b Kaplan–Meier plot of overall survival via the Human Protein Atlas database: patients with renal cancer (a) and endometrial cancer (b) were classified by the ATF5 expression level. c Analysis of differentially expressed genes in shnc versus shATF5 cells by RNA sequencing. d Investigating the expression level of ATF5 in cells treated by normoxia or hypoxia. e, f Investigating the expression (e) and poly-ubiquitin (f) level of HIF1α in identified cells treated by normoxia. Figure S2. a Detection of the expression level of identified proteins in shATF5 vs. shnc cells by western blot in normoxia. b Detection of the expression level of identified mRNAs in shATF5 versus shnc cells by RT-PCR; p < 0.01 in normoxia. c Evaluating the secretion of VEGFA in the identified cells by ELISA in normoxia; p < 0.01. d Dual-luciferase assays were performed to detect the luciferase activation of identified genes in ESCA cells transfected with shnc and shKDM4C in normoxia; p < 0.01. e Investigating the tube formation ability of HUVECs induced by supernatant from medium of identified cells in normoxia; Scale bar 100 μm; p < 0.01. Fgure S3 a, b Examining the interaction between ATF5 and HIF1 target gene promoters by CHIP in normoxia. c, d Investigating the interaction between HIF1 transcriptional complex and VEGFA promoter in shnc and shATF5 cells by CHIP in normoxia; p < 0.01. e Investigating the interaction between ATF5 and HIF1 transcriptional complex on endogenous VEGFA promoters by ChIP/Re-ChIP in normoxia. f Whole-cell extracts of KYSE30 cells were collected for IP analysis using the indicated antibodies, followed by IB analysis in normoxia. g Whole-cell extracts of shATF5 KYSE30 cells were collected for IP analysis using the indicated antibodies, followed by IB analysis in normoxia. Figure S4 a Western blot showing the expression levels of the identified proteins in normoxia. b Dual-luciferase assay showing the activation ability of identified gene [file 12964_2021_734_MOESM3_ESM.docx]
